# Metabolomics profiling in predicting of post-herpetic neuralgia induced by varicella zoster

**DOI:** 10.1038/s41598-023-42363-z

**Published:** 2023-09-11

**Authors:** Lina Lu, Lihong Mei, Xushuo Li, Yanhua Lin, Hongfeng Wang, Gao Yang

**Affiliations:** 1 Department of Dermatology, Jinshan Hospital, Fudan University, Shanghai, 201508 China; 2 Department of Center for Tumor Diagnosis & Therapy, Jinshan Hospital, Fudan University, Shanghai, 201508 China; 3 Department of Clinical Laboratory, Jinshan Hospital, Fudan University, Shanghai, 201508 China

**Keywords:** Metabolomics, Skin diseases

## Abstract

To explore potential metabolomics biomarkers in predicting post-herpetic neuralgia (PHN) induced by herpes zoster (HZ). A total of 90 eligible patients were prospectively enrolled and assigned into an acute pain (ACP) group and a PHN group. Serum samples were collected before clinical intervention to perform metabolomics profiling analyses using gas chromatography mass spectrometry (GC–MS). Key metabolites were identified using partial least squares discriminant analysis (PLS-DA). A binary logistic regression was used to build a combined biomarker model to predict PHN from ACP. The discriminating efficiency of the combined biomarker model was investigated and validated by internal validation. Six metabolites were identified as the key metabolites related to PHN. All these metabolites (*N*-Acetyl-5-hydroxytryptaMine, glucose, dehydroascorbic acid, isopropyl-beta-d-thiogalactopyranoside, 1,5-anhydro-d-sorbitol, and glutamic acid) were found elevated in the PHN group. Pathway analyses showed that glucose-alanine cycle, tryptophan metabolism, tyrosine metabolism, lactose degradation, malate-aspartate shuttle were top five metabolic pathways evolved in PHN. The AUC was 0.85 (95% CI 0.76–0.93) for the combined biomarker model, and was 0.91 (95% CI 0.84–1.00) for the internal validation data set to predict PHN. Metabolomics analyses of key metabolites could be used to predict PHN induced by HZ.

## Introduction

Herpes zoster (HZ) affects about one third of unvaccinated population^1^. The clinical symptoms of HZ is characterized by a painful vesicular rash^2^. Pain is the most frequent complication of HZ, which presents in about 70% of the cases^3^. HZ-induced pain is classified into two categories: acute pain (ACP) and post-herpetic neuralgia (PHN). For ACP, the pain normally resolves over the course of several weeks. For PHN, the chronic pain persists over 90 days after resolution of the rash^4^. PHN is considered as the second most common neuropathic pain after diabetes^5^. PHN is generally distressing and debilitating, which often requires medical intervention^6^.

The pathology of PHN is still unknown. The persistent pain even after the clearance of the virus indicates a damage of the sensory nervous system^7^. Polymerase chain reaction analysis usually fails to detect HZ virus DNA in peripheral blood mononuclear cells of the PHN patients^8^. Previous studies also suggests that central sensitization plays a role in PHN^9^. Currently, the treatment of PHN relies on mitigating symptoms by addressing the chronic pain^7^. Early radical antiviral treatment of HZ could be helpful to prevent the progress of PHN. However, toxicity and side effects should be considered with long term and high dose of antiviral agents^10^. Thus, it is clinical urgency to seek potential biomarkers to predict PHN accurately for PHN management.

Metabolomics can be used to identify biomarkers in differentiating the disease and the control cases through analyzing the changes of metabolites^11^. It can also be used to understand the pathogenesis and metabolic pathway of the diseases. Kuhn et al., reported that metabolomics profiling could be used to identify biomarkers for diagnosis and risk stratification of HZ reactivation^12^. However, to our knowledge, no study on the metabolomics profiling in predicting PHN was reported.

We assumed that metabolomics profiling could be used for predicting PHN induced by HZ. To explore this hypothesis, we used gas chromatography mass spectrometry (GC–MS) to perform a metabolomics profiling analysis to identify potential metabolic biomarkers to provide a new method for early predicting of PHN.

## Results

### Patient baseline characteristics

In total, serum samples of 90 subjects were collected. Of these (median age 64 years; ranged 40–94 years), there were 48 ACP patients (median age 60 years; ranged 40–78 years) and 42 PHN patients (median age 72 years; ranged 44–94 years). The median follow-up time of the ACP patients was 45 days (ranged 21–75 days) and the median follow-up time of the PHN patients was 150 days (ranged 90–720 days). All the patients received Valacyclovir (0.3 g/bid/po) therapy for 7–10 days after serum sampled. The clinical characteristics of all the cases are shown in Table 1.

**Table 1:** Comparison of clinical characteristics between ACP and PHN patients.

	ACP (N = 48)	PHN (N = 42)	P
Gender			1
Female	29 (60.4%)	25 (59.5%)	
Male	19 (39.6%)	17 (40.5%)	
Age (years)	59.3 (10.0)	69.4 (12.5)	< 0.001
Rash duration (days)	10.0 (10.5)	59.1 (54.7)	< 0.001
Rash position			0.387
Face	4 (8.3%)	7 (16.7%)	
Hips	3 (6.3%)	2 (4.8%)	
Lower limb	6 (12.5%)	1 (2.4%)	
Lumbar	4 (8.3%)	1 (2.4%)	
Lumbar/dorsum	14 (29.2%)	11 (26.2%)	
Neck/Shoulder	3 (6.3%)	3 (7.1%)	
Thoracodorsal	13 (27.1%)	14 (33.3%)	
Upper limb	1 (2.1%)	3 (7.1%)	
Pain duration (days)	49.8 (15.7)	188 (119)	< 0.001
Maximum pain time point			< 0.001
1 weeks	33 (68.8%)	9 (21.4%)	
2 weeks	14 (29.2%)	17 (40.5%)	
3 weeks	1 (2.1%)	14 (33.3%)	
4 weeks	0 (0%)	2 (4.8%)	
Rash area			< 0.001
Small	20 (41.7%)	3 (7.1%)	
Median	24 (50.0%)	26 (61.9%)	
Large	4 (8.3%)	13 (31.0%)	
Pain level			0.807
Mild	22 (45.8%)	17 (40.5%)	
Moderate	11 (22.9%)	12 (28.6%)	
Severe	15 (31.3%)	13 (31.0%)	
Onset month			0.263
Feb	0 (0%)	5 (11.9%)	
Mar	0 (0%)	1 (2.4%)	
Apr	3 (6.3%)	3 (7.1%)	
May	11 (22.9%)	9 (21.4%)	
Jun	7 (14.6%)	6 (14.3%)	
Jul	6 (12.5%)	6 (14.3%)	
Aug	0 (0%)	2 (4.8%)	
Sep	13 (27.1%)	6 (14.3%)	
Oct	5 (10.4%)	3 (7.1%)	
Nov	1 (2.1%)	0 (0%)	
Dec	2 (4.2%)	1 (2.4%)	

Data presented as mean (SD) or N (ratio).

**Figure 1:**
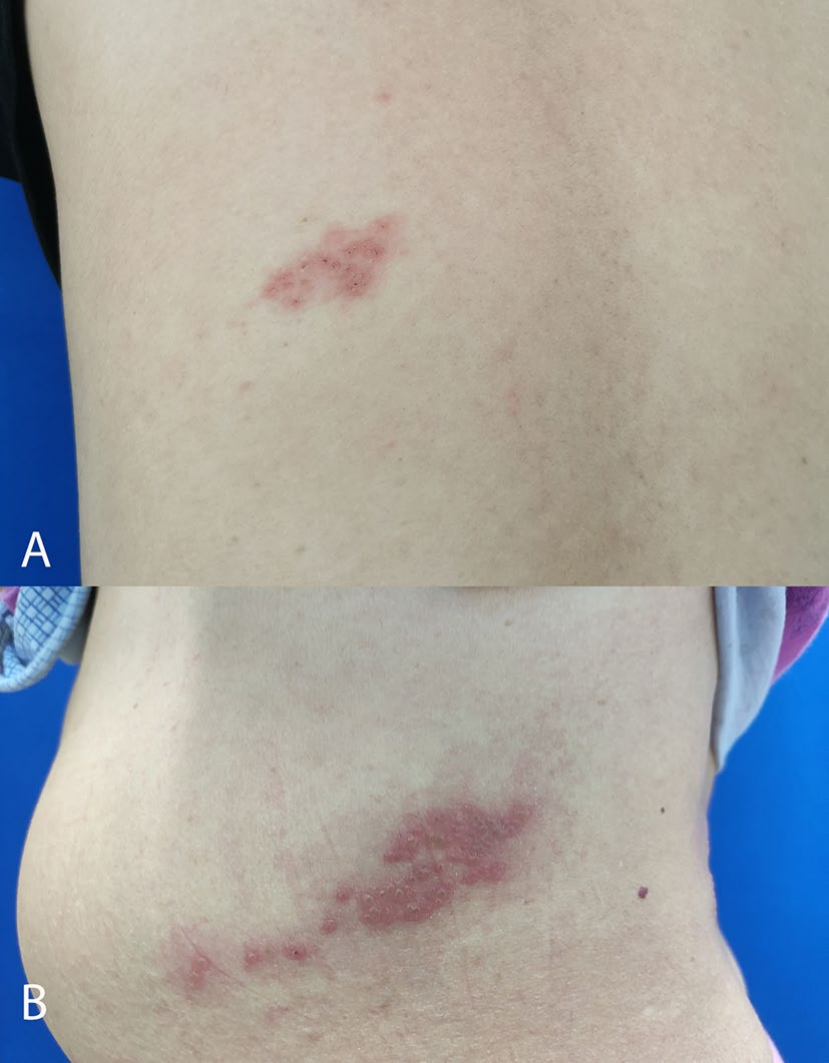
An example of two cases (ACP and PHN). A smaller rash area is seen in a ACP case (Male, aged 35 years) (**A**) A lager rash area is seen ina PHN case (Female, aged 76 years) (**B**).

No significant differences of the gender, rash position, pain level and onset month were shown between two groups. Older age, longer rash duration, longer pain duration, and lager rash area were shown in PHN group compared with ACP group (Table 1). The maximum pain time point was 1 week for PHN group and 2 weeks for ACP group (Table 1). Two cases scenario is exhibited in Fig. 1.

### Metabolomics profiling of PHN serum samples

**Figure 2:**
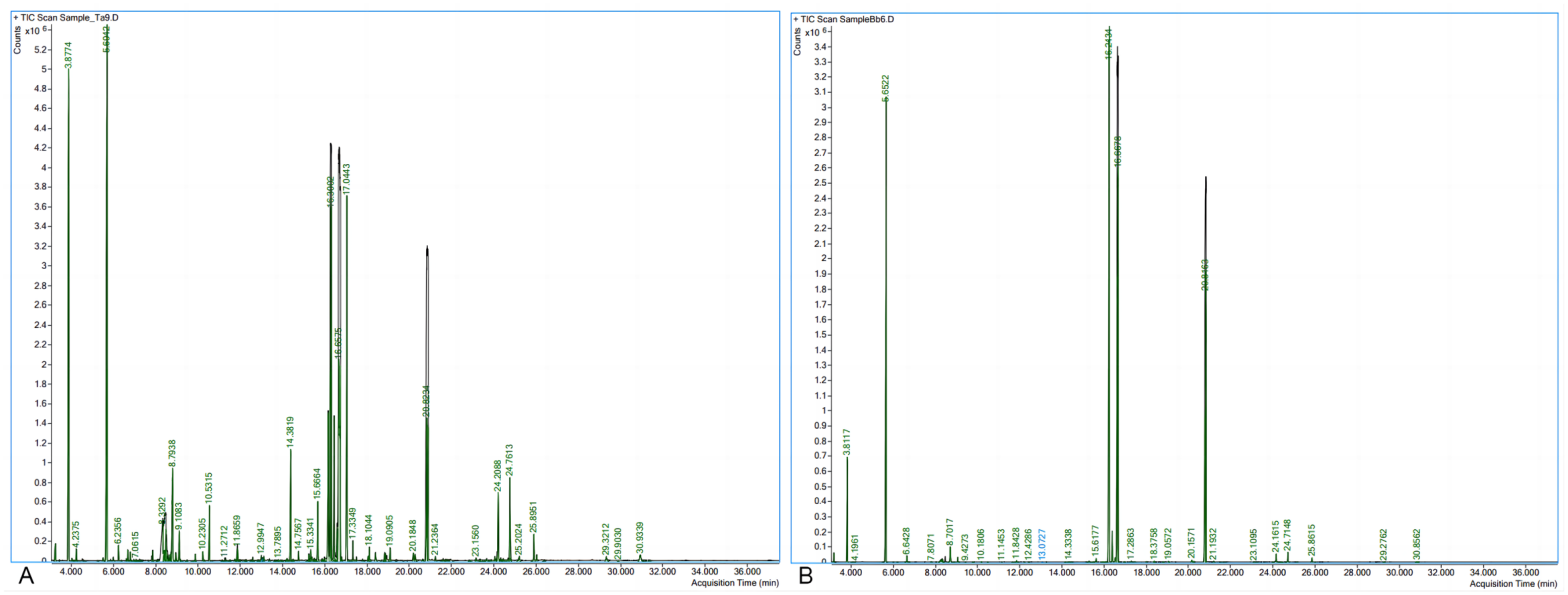
Significant difference of spectral feature is seen in total ion current between ACP group (**A**) and PHN group (**B**).

**Figure 3:**
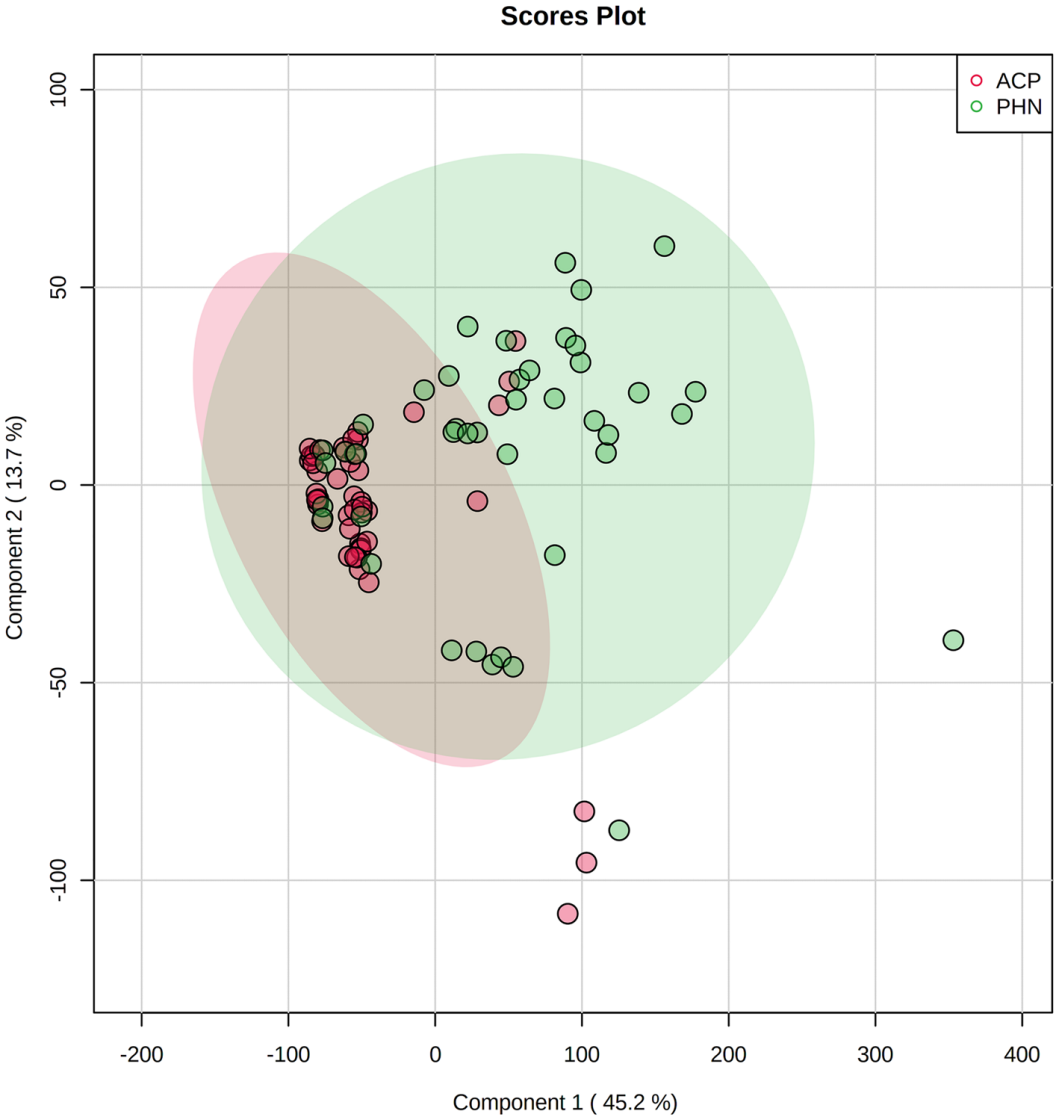
Partial least squares discriminant analysis (PLS-DA) of the metabolites in ACP and PHN groups. The metabolites between ACP and PHN groups can be separated by PLS-DA.

The serum samples were analyzed using GC–MS. A total of 90 metabolites were obtained. The spectral features of total ion current of ACP group and PHN group are shown in Fig. 2. PCA plot shows the metabolites in two groups with QC samples in Supplementary Fig. 1. The metabolites in two groups could be separated by PLS-DA scores plots with accuracy, R^2^, and Q^2^ of 78%, 0.32 and 0.28, respectively (Fig. 3).

**Figure 4:**
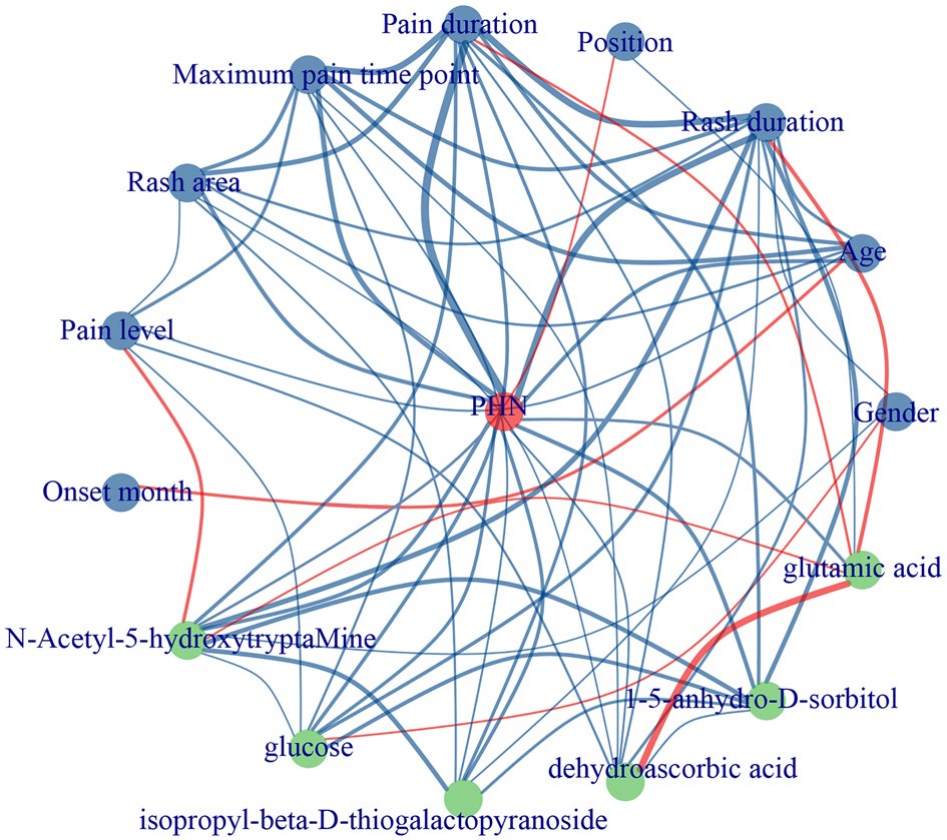
The co-occurrence network shows the correlation of the key metabolites and the clinical features (by Spearman correlation analysis). Significantly correlation is plotted by blue line (positive correlation) and red line (negative correlation). Positive correlations between PHN and age, rash duration, pain duration, maximum pain time point, rash area, pain level, *N*-Acetyl-5-hydroxytryptaMine, glucose, dehydroascorbic acid, isopropyl-beta-d-thiogalactopyranoside, 1,5-anhydro-d-sorbitol, and glutamic acid are seen.

Six metabolites (*N*-Acetyl-5-hydroxytryptaMine, glucose, dehydroascorbic acid, isopropyl-beta-d-thiogalactopyranoside, 1,5-anhydro-d-sorbitol, and glutamic acid) with VIP > 1, FDR < 0.05, and AUC > 0.6 were identified. These metabolites were considered as the potential biomarkers for predicting PHN induced by HZ. A co-occurrence network shows the correlation of the key metabolites and the clinical features (Fig. 4).

The fold change, VIP, FDR, and AUC for the key metabolites is shown in Table 2. Significant up-regulated of *N*-Acetyl-5-hydroxytryptaMine, glucose, dehydroascorbic acid, isopropyl-beta-d-thiogalactopyranoside, 1,5-anhydro-d-sorbitol, and glutamic acid were found in PHN patients. The AUC was 0.85 (95% CI 0.76–0.93) for the combined biomarker model with specificity, sensitivity and accuracy of 71%, 85% and 78%, respectively. For the internal validation data set, the AUC was 0.91 (95% CI 0.84–1.00) with specificity, sensitivity and accuracy of 75%, 93% and 83%, respectively.

**Table 2:** The VIP, fold change, and FDR of the six key metabolites.

ID	FC	FDR	VIP	AUC	95% CI
*N*-Acetyl-5-hydroxytryptaMine	4.36	< 0.001	3.7	0.74	0.60–0.82
Glucose	4.46	< 0.001	3.6	0.71	0.60–0.82
Dehydroascorbic acid	50.80	0.0001	3.5	0.72	0.61–0.82
Isopropyl-beta-d-thiogalactopyranoside	2.49	0.0004	2.3	0.63	0.50–0.75
1,5-Anhydro-d-sorbitol	4.58	< 0.001	1.8	0.75	0.65–0.86
Glutamic acid	62.91	0.0064	1.7	0.65	0.53–0.79

*AUC* area under the receiver operator characteristic curve, *FC* fold change (PHN/ACP), *FDR* false discovery rate, *VIP* influence on projection.

### Enrichment and pathway analysis

**Figure 5:**
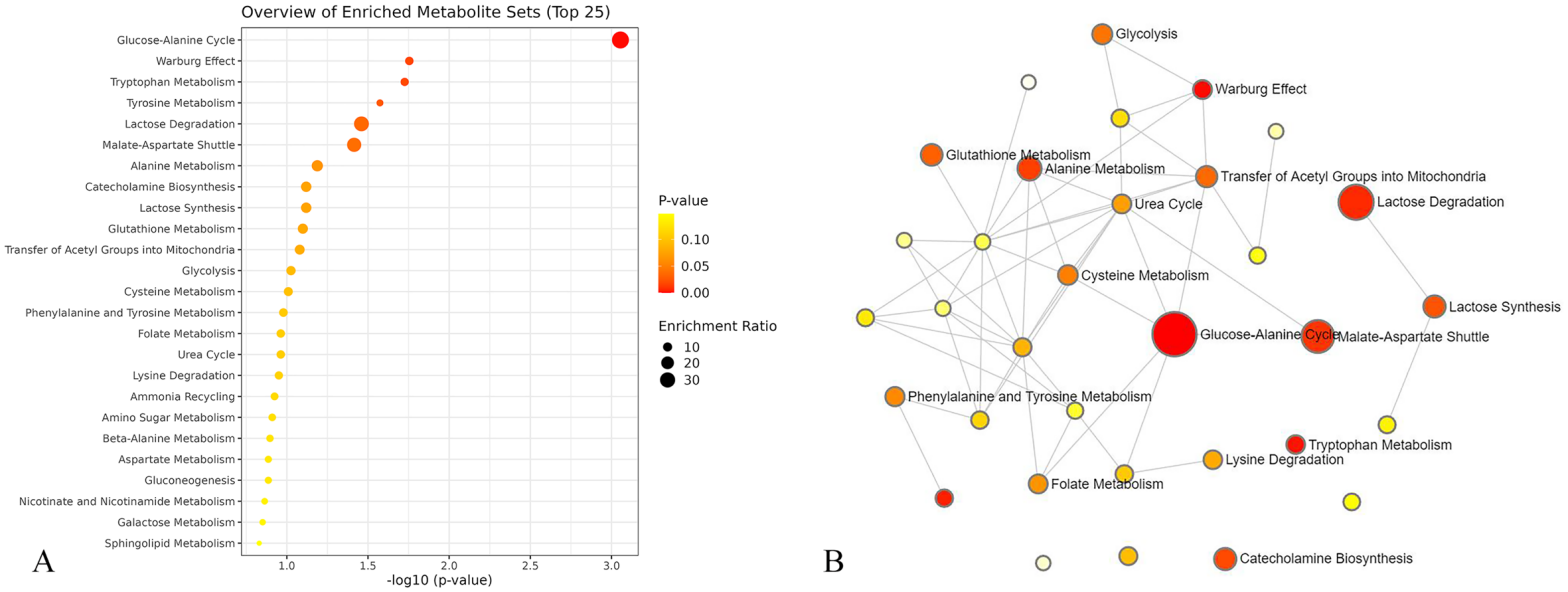
Metabolic pathways analysis of the metabolites. Glucose-alanine cycle, tryptophan metabolism, tyrosine metabolism, lactose degradation, malate-aspartate shuttle are top five metabolic pathways evolved in PHN induced by HZ (**A**,**B**).

Enrichment and pathway analysis showed that glucose-alanine cycle, tryptophan metabolism, tyrosine metabolism, lactose degradation, malate-aspartate shuttle were top five metabolic pathways evolved in PHN (Fig. 5).

## Discussion

In this study, GC–MS metabolomics was used for predicting PHN induced by HZ. By using metabolomics analysis, six metabolites were identified as the key metabolites involved in the PHN metabolism.

ACP was involved in almost all the HZ cases, of these, it was reported that about 10%-35% cases could progress to PHN^13^. The transition from acute pain to chronic pain is poorly understood. Previous studies indicated that larger rash, greater acute pain, and weakened immunity were correlated with the development of PHN^6^. In this study, older age, longer rash duration, longer pain duration, longer maximum pain time point, and lager rash area were correlated with PHN. The result was in accordance with the previous reports.

Previous studies indicated that metabolomics can provide candidates of potential biomarkers of diseases^14,15^. Increasing of glutamic acid levels was reported in multiple metabolomics studies in neuropathic pain conditions^16^. Alexander et al. found that change in plasma glutamic acid was association with the condition of complex pain syndrome^17^. In this study, higher serum glutamic acid were found in PHN patients. The stimulation caused by glutamic acid may induce hyperalgesia in chronic pain.

Ramautar et al. reported that significantly different metabolic profiles were identified in patients with chronic pain^18^. Significantly increased 2-ketoisovalerate, glucose, glutamine, and lactate, and significantly decreased urea concentration were reported. The results suggests that chronic pain is associated with carbohydrate metabolism, which is likely associated with inflammation. Meissner et al. reported that increased 2-aminobenzoic acid and decreased creatine were found in patients with chronic pain^19^. Slimier result was also reported in an animal model of peripheral nerve injury-induced neuropathic pain^20^. These results suggest that amino acid metabolism and carbohydrate metabolism are involved in chronic pain in^21^. In this study, elevated serum glucose, isopropyl-beta-d-thiogalactopyranoside and 1,5-anhydro-d-sorbitol were found in PHN patients. The result indicated an impaired carbohydrate metabolism in PHN patients, which was in accordance with the previous reports.

5-hydroxytryptamine (5-HT) plays an important role in the occurrence and development of central and peripheral pain^22^. In central nervous system, as a neurotransmitter, 5-HT participates in the regulation of pain, sleep, and emotions. In peripheral system, 5-HT is released by platelets and mast cell in the blood, which would aggravate the pain^22^. In this study, elevated serum *N*-Acetyl-5-hydroxytryptaMine reflected an elevation of 5-HT in the progress of chronic pain. The ascorbic acid (vitamin C)/dehydroascorbic acid system reflects the oxidative stress status of the system. Previous studies supported the potential role of vitamin C in the management of chronic pelvic pain^23^. An elevated serum dehydroascorbic acid suggested the presence of oxidative stress in PHN patients, which might be related to the chronic pain.

There are some limitations of this study. First, metabolites are sensitive to many factors, which resulting in high variability of it. Small samples may lead to bias in the key metabolites selection. In this study, a strict selection was used to prevent overfitting. More key metabolites might be discovered in prospective studies from multi-center with larger samples. Second, the results of this study need further validation to ensure consistency and repeatability. Last, more reliable evidence based on the mechanistic studies should be provided before clinical application.

In conclusion, the high accuracy of metabolomics profiling provided a possibility to predict PHN induced by HZ.

## Methods

### Study design and patient population

This prospective study was approved by the Institutional Review Board of Jinshan Hospital, Fudan University (No. JIEC2021S18). All the experiments were performed in accordance with relevant guidelines and regulations. All subjects were informed the purpose of the study and signed the informed consent form before sample collection.

From April 2021 to April 2022, 90 consecutive HZ patients were enrolled in this study. All these patients were infected with varicella zoster virus, and they received a evaluation of pain by numerical rating scale. Exclusion criteria: (1) patient with complicated with autoimmune diseases (such as systemic lupus erythematosus, Sjogren's syndrome, rheumatoid arthritis); (2) patient with malignant tumor or with other course of acute or chronic pain; (3) patient with severe liver, kidney, cardio cerebrovascular diseases; (4) patient with long term using of hormones or immunosuppressants; (5) patient with severe infection in other parts and/or those unable to evaluate pain. The patients were followed up every week until pain disappears or significantly alleviates. According to the pain duration, these patients were assigned into an ACP group (pain duration < 90 days) and a PHN group (pain duration ≥ 90 days).

### Clinical features and serum sample collection

Clinical features including age, gender, rash duration, rash position, pain duration, maximum pain time point, rash area, pain level and onset month were recorded. The patients were clinically examined by two physicians. Fasting peripheral blood was collected with a serum separator tube before clinical intervention.

### Metabolite extraction and profiling analysis

The chemicals and reagents were subscribed from Fisher Chemical (Thermo Fisher, USA) or Sigma-Aldrich (St. Louis, MO, USA), including water, methanol, pyridine, acetone, ammonium hydroxide solution, ammonium acetate (NH_4_OAc), methoxyamine hydrochloride, and *N*-methyl-*N*-(trimethylsilyl) trifluoroacetamide (MSTFA).

For metabolites extraction, 100 uL of each sample was used. Quality control (QC) samples were prepared by combining every ten samples. 400 uL solution (methanol:water = 4:1) was added to the samples. The mixtures were centrifuged at 12,000 rpm for 15 min at 4 °C. The supernatants were collected and concentrated and freeze-dried for 3 h in V-AQ mode. The freeze-dried samples were resuspended in 50 μL methoxyamine hydrochloride with pyridine (20 mg/mL). The mixtures were vortexed and incubated for 90 min at 30 °C to form methoxyamine derivatives. Subsequently, 40 μL of MSTFA was added to the samples for silylation reaction and incubated for 30 min at 37 °C. The supernatants were collected after centrifugation at 14,000 rpm for 5 min for further GC–MS analysis.

Untargeted metabolomics analysis was carried out by GC–MS using an Agilent 7890B GC with 5977B inert mass selective detector (MSD) system (Agilent Technologies, Santa Clara, CA, USA). GC–MS data were collected on the GC/MSD system operating in election ionization mode (70 eV) and selected ion monitoring mode. After column heating procedure (starting temperature 80 °C for 2 min, 10 °C/min to 180 °C, 5 °C/min to 240 °C, 25 °C/min to 290 °C for 9 min), the samples were injected in the GC/MS in splitless mode with inlet temperature set to 270 °C. The helium was used as a carrier gas with a flow rate of 1 mL/min. The quadrupole was set at 150 °C and the GC/MS interface was set at 260 °C. The oven program for all metabolite analyses started at 60 °C held for 1 min, then increasing at a rate of 10 °C/min until 200 °C. Bake-out was set at 200 °C for 5 min. Data were acquired both in scan (30–600 m/z) and selected ion monitoring modes.

The raw data from GC–MS was processed using Agilent MassHunter Qualitative Analysis software (version 10.0, Agilent, CA, USA). The ion intensities were integrated and then compared and matched with the Agilent Fiehn database. The metabolites were identified according to the mass spectrum matching degree. All metabolites identified in this study were previously validated using authentic standards to confirm mass spectra and retention times.

### Data processing

First, metabolites with at least 80% samples in each group were retained. Second, the remaining missing values were replace by median. Third, the data was normalized by median and scaled by Pareto scaling (mean-centered and divided by the square root of the standard deviation of each variable).

Unsupervised analysis of principal component analysis (PCA) was firstly performed to explore the metabolites to assess for sample similarities between ACP and PHN groups. The clustering patterns and outliers were explored. Then, supervised analysis of projection to latent structures discriminant analysis (PLS-DA) was performed to build more discrimination between ACP and PHN groups by calculating the variable influence on projection (VIP) score. Third, the area under receiver operator characteristic (ROC) curve (AUC) were employed to complement the PLS-DA classification performance in predicting PHN. The metabolites with VIP score > 1, false discovery rate (FDR) < 0.05, and AUC > 0.6 were defined as the key metabolites. A co-occurrence network was computed to analyze the correlation between the metabolites and the clinical features by Spearman correlation analysis. A binary logistic regression was used to construct a combined biomarker model in predicting PHN by combining the key metabolites to improve the discriminating efficiency.

One third of all the cases were selected randomly to form an internal validation data set. The discriminating efficiency of the combined biomarker model was validated.

### Enrichment and pathway analysis

Enrichment and pathway analysis of the key metabolites were performed based on the KEGG database (https://www.kegg.jp/). The top-ranked pathways related to the PHN were explored.

### Statistical analysis

MetaboAnalyst (v5.0, https://www.metaboanalyst.ca/) and R (v4.2.0, https://www.r-project.org/) were used to perform the statistical analyses. Data met normality and variance homogeneity were analyzed by T-test followed by false discovery rate (FDR) correction. If not met normality or variance homogeneity, the data were analyzed by Mann–Whitney U test followed by FDR. P < 0.05 was considered statistically significant.

### Supplementary Information

Supplementary Figure 1.

## Data Availability

The datasets generated during and/or analyzed during the current study are available from the corresponding author on reasonable request.
