# Supplementary figures and images for: Metabolomics profiling in predicting of post-herpetic neuralgia induced by varicella zoster
Source: Sci Rep. 2023 Sep 11;13:14940. doi: 10.1038/s41598-023-42363-z (PMC10495364; doi:10.1038/s41598-023-42363-z)

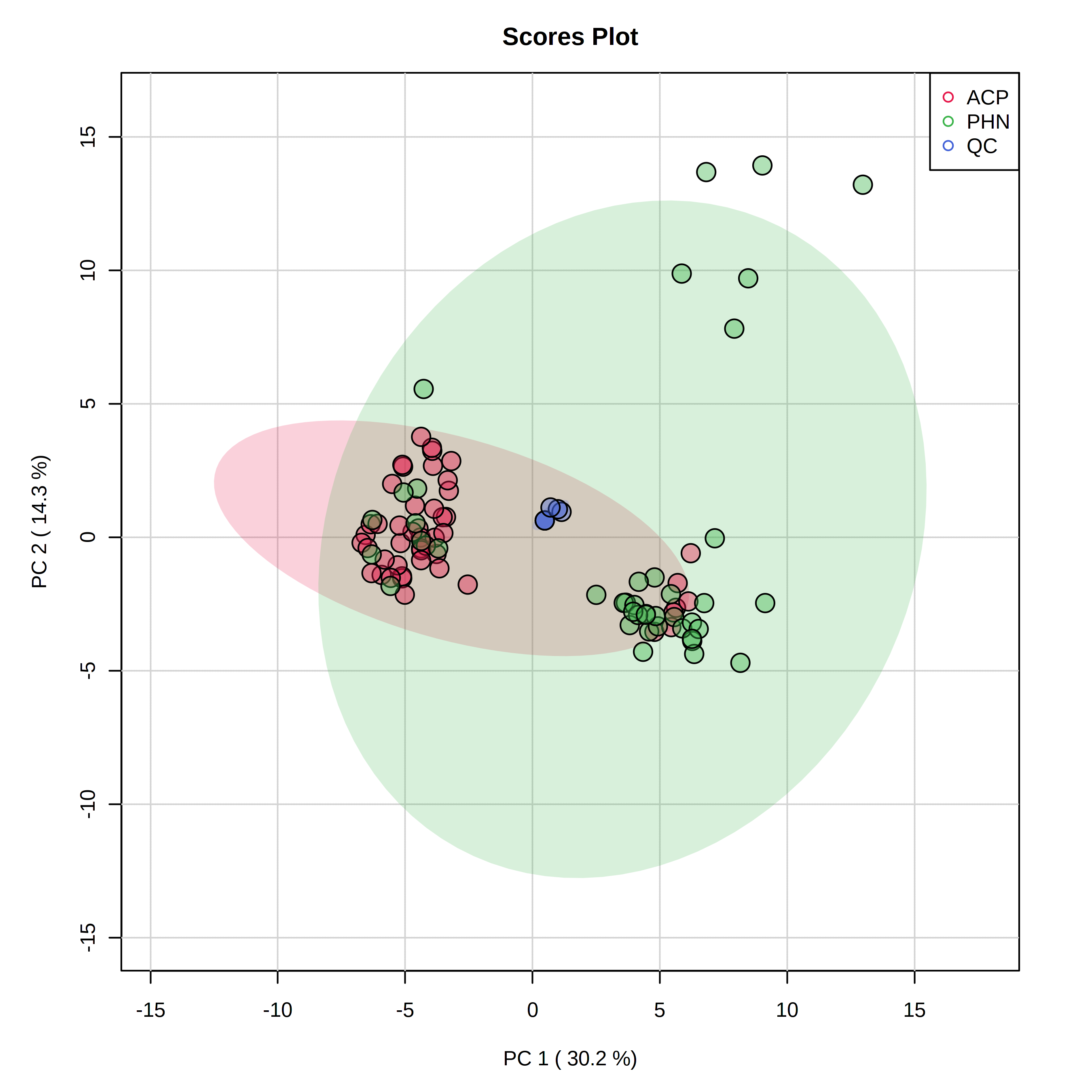


Supplementary Figure 1. principal component analysis plot of the ACP and PHN with QC samples.

Supplement: Supplementary file 1 — Supplementary Figure 1. [file 41598_2023_42363_MOESM1_ESM.docx]
